# A case of wedge resection of duodenum for massive gastrointestinal bleeding due to duodenal metastasis by renal cell carcinoma

**DOI:** 10.1186/1477-7819-10-199

**Published:** 2012-09-25

**Authors:** Hongzhi Zhao, Keqiang Han, Jing Li, Ping Liang, Guohua Zuo, Yu Zhang, Hongyan Li

**Affiliations:** 1 Department of Hepatobiliary Surgery, Xinqiao Hospital, Third Military Medical University, Xinqiao Street, Chongqing 400037, China; 2 Division of Immunology &Inflammation, Department of Medicine, Imperial College, Hammersmith Campus, Du Cane Road, London, W12 0NN, UK

**Keywords:** Gastrointestinal bleeding, Wedge resection, Metastasis, Renal clear cell carcinoma

## Abstract

**Background:**

Gastrointestinal bleeding due to duodenal metastasis from renal cell carcinoma is extremely rare. Several previous reports have shown that embolic therapy or pancreatoduodenectomy (radical surgical resection) could be effective in controlling this type of clinical complication. Management is entirely dependent on the general condition and concurrent metastases at other sites. Optimizing the therapeutic strategies thus deserves further discussion and exploration.

**Methods:**

In this report, we describe a patient with severe co-morbidities who underwent successful palliative wedge resection of duodenum and direct duodenal wall defect repair without reconstruction of duodeno-jejunostomy for acute upper digestive tract hemorrhage caused by duodenal metastasis from renal clear cell carcinoma.

**Results:**

The patient recovered uneventfully and did not experience rebleeding and frequent vomiting after surgery. Since then (1.5 years) he has had no evidence of rebleeding.

**Conclusions:**

Gastrointestinal bleeding due to duodenal metastasis of RCC may benefit from emergent resection even in the presence of severe co-morbidities, and for palliative treatment.

## Background

Renal cell carcinoma (RCC) accounts for 3% of all adult malignancies, and is the third most frequent urologic malignancy after prostate and bladder cancer [1]. Additionally, nearly 25% to 50% will develop metastatic disease metachronously after surgical treatment of the primary renal mass [2]. While the most common sites of metastasis are the lung, bone, liver, adrenal, and brain, some unusual sites have also been reported including the iris, thyroid, breast, urinary bladder, epididymis, small bowel, pancreas, spleen, gallbladder, and ampulla [3,4]. Acute upper gastrointestinal hemorrhage due to duodenal metastasis from RCC is a rare event. To the best of our knowledge, there have been a few reports in which embolic therapy or pancreatoduodenectomy have been employed to stop bleeding from RCC duodenal metastasis. Both methods are proved to be useful in controlling upper gastrointestinal bleeding from this cause [2,5]. Embolization is a less invasive surgery but the RCC metastasis may re-bleed after treatment. On the other hand pancreatoduodenectomy offers control of bleeding and cure of duodenal metastasis but in these patients morbidities from the procedure may be excessive. In other words, such surgical therapy could not only stop bleeding but also remove the duodenal metastatic tumor, in spite of high risk of morbidity especially for those patients suffering from cachexia to go through the surgical procedure. Here, we present a case of successful management of duodenal bleeding caused by metastasis from RCC by a wedge resection of duodenum with an excellent long-term outcome.

## Methods

### Preoperative diagnostics and medical history

A 56-year-old man was referred to us with a diagnosis of presumed duodenal carcinoma.

The patient had undergone right nephrectomy in 2005 for renal clear cell carcinoma (pT2, pV0, pN0: stage II). The postoperative course was uneventful and no adjuvant therapy was given. During the 5-year follow-up, fecal occult blood test had been carried out as a routine test. No signs of tumor recurrence were detected during the follow-up with annual abdominal ultrasonography, and the physical examination was unremarkable.

**Figure 1:**
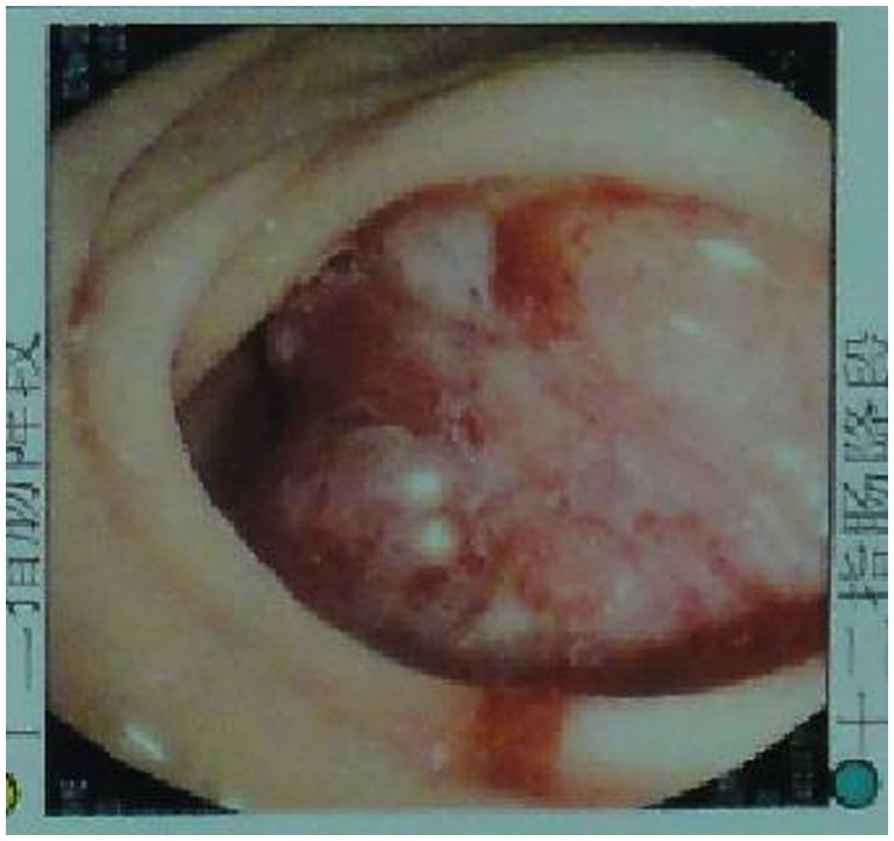
**Gastroscopy showing a mass in the descending portion of the duodenum with mucosal ulcerations and focal hemorrhage.** The whole lumen of the duodenum was occupied by the tumor, and the duodenal papilla cannot be visualized.

**Figure 2:**
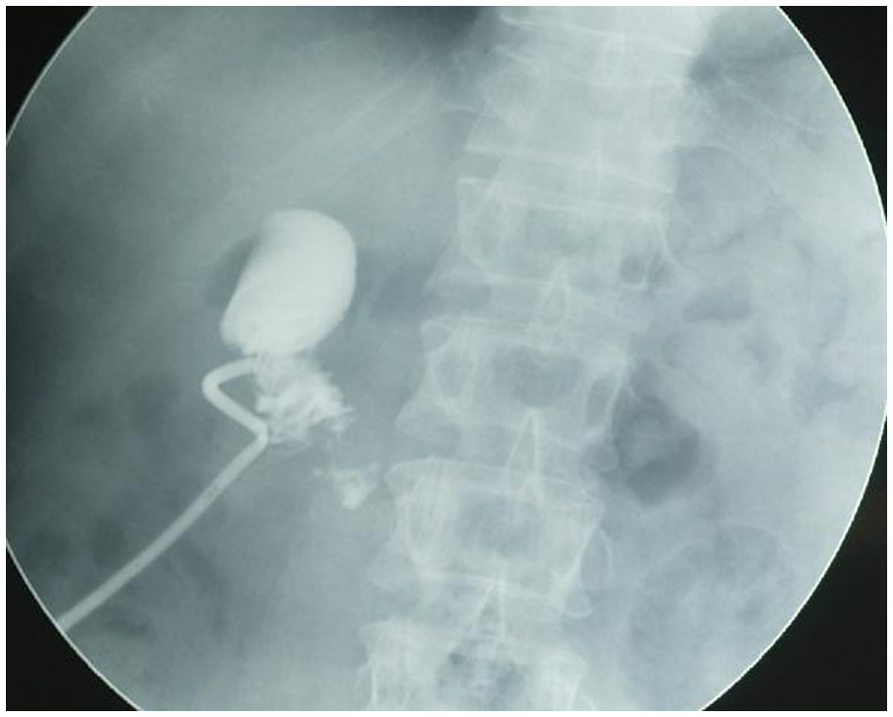
**Upper GI meal barium showing a filling-defect in the descending and the horizontal portion of the duodenum.** The mucous membrane was not smooth, and there was limited dilatation.

**Figure 3:**
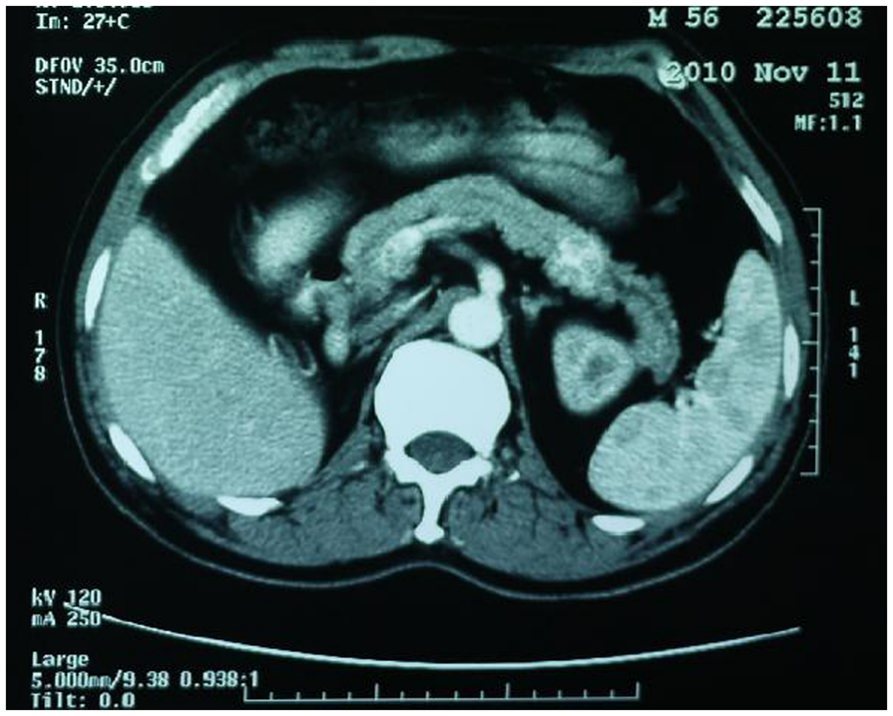
**Abdominal computed tomography showed a 2.0 cm enhancing mass in the pancreatic tail.** According to CT arterial phase, the pancreatic mass revealed enhancing lesions.

The patient was admitted by the department of gastroenterology. The main complaints were generalized fatigue, continuous melena, and frequent vomiting for 20 days. These symptoms were not relieved by using medications and supportive care (like fluids, parenteral nutrition, and blood transfusion). For further treatment, after 20 days admission, the patient was transferred to the department of hepatobiliary surgery. Peripheral blood cell counts demonstrated severe anemia and a hemoglobin level of (54 g/L). Blood analyses revealed hypoproteinemia (44 g/L) with hypoalbuminemia (25 g/L). Other laboratory examinations such as blood chemistry and serum tumor markers were normal. Gastroscopy showed a mass in the descending part of the duodenum with mucosal ulcerations and focal hemorrhage. The whole lumen of the duodenum was occupied by the mass, and the duodenal papilla could not be visualized (Figure 1). A duodenal biopsy was performed and histopathology diagnosis suggested adenocarcinoma of the duodenum. An upper GI series showed a filling-defect in the same area (Figure 2), and an abdominal computed tomography (CT) confirmed the presence of a 2.5-cm filling-defect. Another lesion 2.0 cm in diameter was detected in the pancreatic tail (Figure 3). Preoperative clinical diagnostic evaluation resulted in the diagnosis of a massive gastrointestinal bleeding, duodenal carcinoma with incomplete duodenal obstruction, pancreatic tail carcinoma, severe anemia, hypoalbuminemia, renal cell carcinoma status post right nephrectomy.

### Surgical therapy

**Figure 4:**
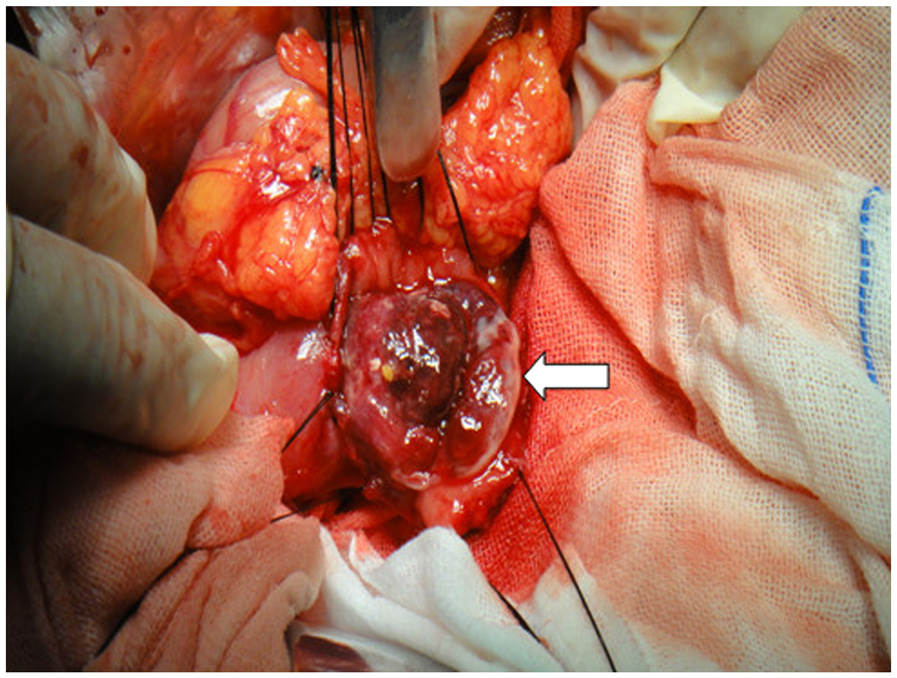
**Tumor invasion of the descending duodenum with mucosal ulcerations and focal hemorrhage.** The ampulla of Vater was not affected, but part of the duodenum was tightly adherent to the surrounding tissue, including the inferior vena cava and retroperitoneal space.

An exploratory laparotomy was carried out through a median laparotomy incision. A standard Kocher maneuver opening the gastrocolic ligament was performed to assess the duodenum and the extent of lesion. The lateral wall of the descending part of duodenum was longitudinally opened to visualize the ampulla of Vater and protect it during resection of the lesion. A lesion was identified that invaded the descending part of duodenum wall but not the ampulla of Vater. Part of the lesion was tightly adhered to the surrounding tissue, including part of the inferior vena cava and retroperitoneal space (Figure 4). After completely separating it from the inferior vena cava and retroperitoneal space, the lesion with part of duodenum was finally removed from duodenum to complete the resection. A full-thickness wedge resection and direct duodenal wall defect repair were performed, but without reconstruction of duodeno-jejunostomy. The intraoperative frozen sections confirmed the lesion of pancreatic tail and duodenum metastasis from RCC. According to the results of laparotomy and preoperative CT, if the lesion on the pancreatic tail was resected, the spleen would also have to be removed. In general, if anemia and hypoproteinemia had been effectively corrected, resection of duodenal and pancreatic tail, or even spleen resection, would have been feasible. Considering the patient’s poor general condition (severe anemia, hypoalbuminemia), tumor location and involvement, a local resection via duodenostomy was performed and the lesion in the pancreatic tail was left untreated for saving the patient’s life as the first priority.

## Results and discussion

### Postoperative clinical course and histopathology

**Figure 5:**
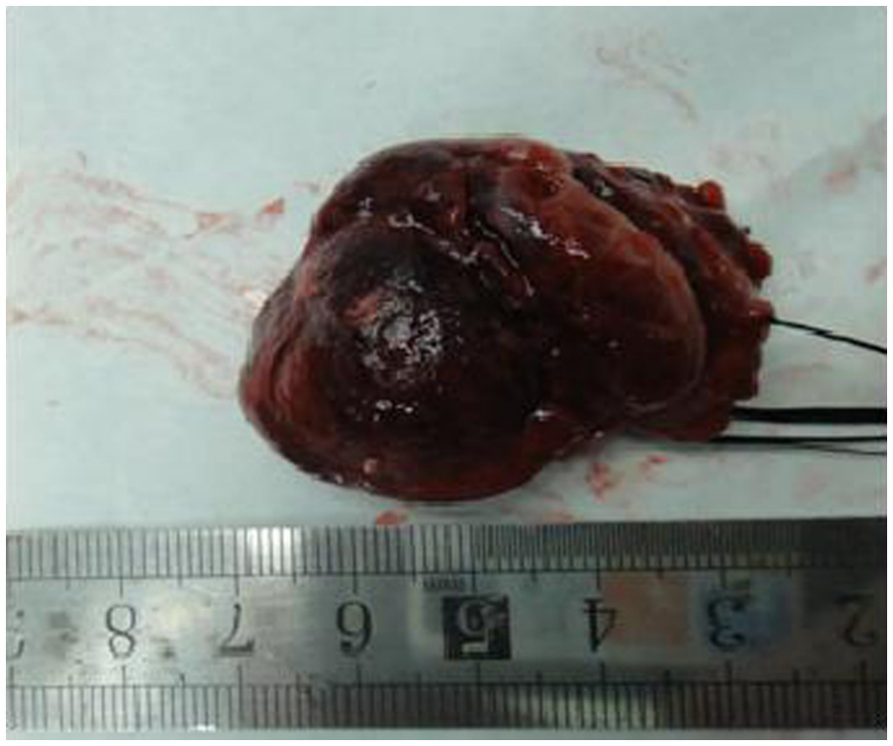
Gross appearance of the resected tumor.

**Figure 6:**
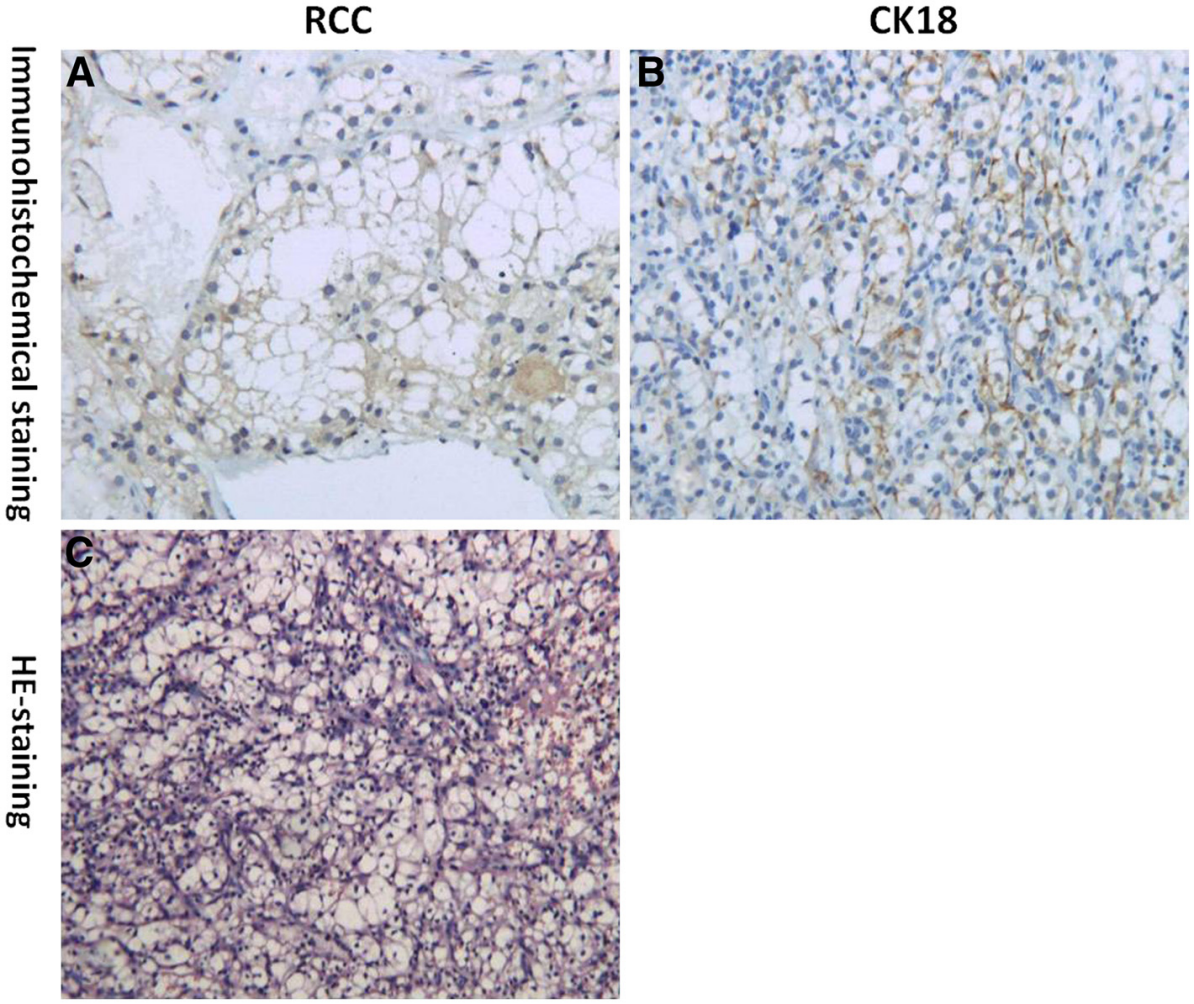
**(A) Immunohistochemical staining by renal cell carcinoma (RCC) (original magnification, ×400).** (**B**) Immunohistochemical staining by Cytokeratin 18 (CK18) (original magnification, ×400). (**C**) Hematoxylin and eosin (HE)-staining showing polygonal cells with clear cytoplasm and relatively uniform nuclei, some of them exhibiting prominent nucleoli (original magnification, ×400).

Pathologic findings revealed a 4.3-cm mass in the duodenal that appeared to be a clear cell carcinoma of renal origin (Figure 5). Immunohistochemistry of the mass revealed strong and diffuse expression of cytokeratin 18 and RCC in tumor clear cells (Figure 6A, 6B). Immunostaining for CD117, vimentin, and EGFR were uniformly negative in tumor cells. Upon microscopic examination of hematoxylin and eosin (HE)-stained tissue sections, the duodenal mass was found to be populated by polygonal cells with clear cytoplasm and relatively uniform nuclei, some of which exhibited prominent nucleoli (Figure 6C). Based on the above information, a diagnosis of duodenal and pancreatic tail metastasis by renal clear cell carcinoma was made. The patient recovered uneventfully and did not experience rebleeding and frequent vomiting after surgery. Since then (1.5 years) he has had no evidence of rebleeding.

### Discussion

RCC metastasis to the duodenum is a rare event, accounting for 7.1% of all small bowel metastases [6,7]. Duodenal metastasis from RCC may present with abdominal pain, nausea, weight loss, jaundice, anemia, gastrointestinal bleeding, duodenal obstruction, perforation and duodenal intussusceptions [8]. It can occur at any time after nephrectomy [9], and is indistinguishable from other gastrointestinal diseases. If there are specific mucosal changes in the duodenal lumen, the diagnosis can be made by gastroscopy. The diagnosis should be considered in any patient with upper gastrointestinal bleeding or obstructive symptoms and with right-sided renal tumor or radical nephrectomy in the past [10] (even if metastasis of renal cell carcinoma cannot be definitely diagnosed in routine duodenal biopsy at gastroscopy). There have been several reports on the use of stanniocalcin 2 (STC2) as a marker for the diagnosis of duodenal metastasis from RCC [9]. Results from these studies indicate that STC2 may indeed be useful in determining the postoperative risk stratification of those patients.

Up to now there have been several similar cases reporting acute upper gastrointestinal hemorrhage due to duodenal metastasis from RCC. Currently management is entirely dependent on the general condition and concurrent metastases at other sites [8]. Although immediate life-saving treatment requires emergency arteriography and arterial embolization [5], complications including rebleeding, gastrointestinal ischemia, and non-target embolization are relatively encountered [8]. Moreover, the duodenal obstruction is not solved by using embolization at all. Additionally, the patient was admitted by the department of gastroenterology for 20 days. For the reason of incomplete duodenal obstruction, the patient could not eat food, even in a liquid form. Anemia, hypoproteinemia, and frequent vomiting were not relieved by using medications and supportive care (like fluids, parenteral nutrition, and blood transfusion). The family of the patient could not continue to bear the economic burden and even prepared to abandon the treatment. According to the patient’s clinical conditions (gastrointestinal bleeding with incomplete duodenal obstruction) and economic situation, emergent surgical intervention to stop bleeding and relieve obstruction of the duodenum is the preferred choice. In terms of the surgical treatment of duodenal carcinoma, the most conventional and effective surgical method is pancreatoduodenectomy. Surgical resection of solitary metastatic renal cell carcinoma has resulted in 5-year survival rates from 35% to 50%, and a 5-year disease-free survival rate of 5% to 23% [11]. However, because of the complications and surgical massive trauma associated with this procedure and the poor condition of the patient, a less aggressive and palliative treatment was selected without treating the pancreatic tail, in order to minimize the complications and improve the quality of life. Therefore, a segmental, palliative full-thickness wedge resection of duodenum was emergent undertaken. This procedure can be performed safely if basic principles are followed:

1) A Kocher maneuver is performed to assess the anatomic relationship between the lesion and the bile duct and head of the pancreas. The ampulla of Vater is carefully identified and preserved during the resection process.

2) The lesion is carefully dissected away from the inferior vena cava and retroperitoneal structures. Vascular anastomotic devices and an adequate supply of blood should be present.

3) Clear surgical margins of 1 or 2 cm are obtained [12].

4) A longitudinal incision and transverse suturing of the duodenum is performed to prevent stricture of the duodenum.

5) A patent distal jejunum is present.

## Conclusions

In conclusion, gastrointestinal bleeding due to duodenal metastasis of RCC may benefit from emergent resection even in the presence of severe co-morbidities (severe anemia, hypoalbuminemia, and incomplete duodenal obstruction), and for palliative treatment.

## Consent

Written informed consent was obtained from the patient for publication of this case report and any accompanying images. A copy of the written consent is available for review by the Editor-in-Chief of this journal.

## Abbreviations

CT: Computed tomography; EGFR: Epidermal growth factor receptor; GI: Gastrointestinal; RCC: Renal cell carcinoma; STC2: Stanniocalcin 2.

## Competing interests

The authors declare they have no competing interests. The authors have no conflict of interest to disclose which may have biased their work. The authors declare that they did not receive any funding for this work.

## Authors’ contributions

HZZ operated on the patient, collected the majority of relevant clinical data, and drafted the manuscript. KQH researched the literature for this case and contributed significantly with figures and relevant intellectual comments on the manuscript. JL operated on the patient, collected the majority of relevant clinical data, and helped significantly with the writing of the manuscript. PL had the idea to write this case report, participated significantly in its design and coordination, and helped to improve the intellectual content of the manuscript. GHZ provided relevant surgical experience and advice and helped to improve the intellectual content of the manuscript. YZ operated on the patient, collected the part of relevant clinical data. HYL carried out the histological examination of the resected specimen, including immunohistochemistry, and helped with the writing of the manuscript. All authors read and approved the final manuscript.

## Authors’ information

All authors of this article are clinically highly experienced surgeons who are regularly involved with interdisciplinary treatment decisions for patients with massive gastrointestinal bleeding due to cancer metastasis or post-hepatitis liver cirrhosis, encompassing up-to-date interventional, chemotherapeutic, and surgical approaches, in one of the largest department of hepatobiliary surgery in southwest of China.
